# Liver Fibrosis: Mechanistic Concepts and Therapeutic Perspectives

**DOI:** 10.3390/cells9040875

**Published:** 2020-04-03

**Authors:** Natascha Roehlen, Emilie Crouchet, Thomas F. Baumert

**Affiliations:** 1 Université de Strasbourg, 67000 Strasbourg, France; natascha.roehlen@etu.unistra.fr (N.R.); ecrouchet@unistra.fr (E.C.); 2 Institut de Recherche sur les Maladies Virales et Hépatiques U1110, 67000 Strasbourg, France; 3 Pôle Hepato-digestif, Institut Hopitalo-Universitaire, Hôpitaux Universitaires de Strasbourg, 67000 Strasbourg, France

**Keywords:** Hepatic stellate cell, liver myofibroblast, Kupffer cell, liver cirrhosis, anti-fibrotics, TGF-β, PDGF

## Abstract

Liver fibrosis due to viral or metabolic chronic liver diseases is a major challenge of global health. Correlating with liver disease progression, fibrosis is a key factor for liver disease outcome and risk of hepatocellular carcinoma (HCC). Despite different mechanism of primary liver injury and disease-specific cell responses, the progression of fibrotic liver disease follows shared patterns across the main liver disease etiologies. Scientific discoveries within the last decade have transformed the understanding of the mechanisms of liver fibrosis. Removal or elimination of the causative agent such as control or cure of viral infection has shown that liver fibrosis is reversible. However, reversal often occurs too slowly or too infrequent to avoid life-threatening complications particularly in advanced fibrosis. Thus, there is a huge unmet medical need for anti-fibrotic therapies to prevent liver disease progression and HCC development. However, while many anti-fibrotic candidate agents have shown robust effects in experimental animal models, their anti-fibrotic effects in clinical trials have been limited or absent. Thus, no approved therapy exists for liver fibrosis. In this review we summarize cellular drivers and molecular mechanisms of fibrogenesis in chronic liver diseases and discuss their impact for the development of urgently needed anti-fibrotic therapies.

## 1. Introduction

Chronic liver diseases are a major global health burden and account for approximately 2 million deaths per year worldwide [1]. Underlying etiologies in chronic liver disease comprise viral (Hepatitis B; HBV and hepatitis C; HCV) related chronic liver disease, alcoholic steatohepatitis (ASH), and non-alcoholic steatohepatitis (NASH), as well as autoimmune and genetic diseases. Organ fibrosis characterizes disease progression in chronic inflammatory diseases and contributes to 45% of all-cause mortality world-wide [2]. Similarly, in the liver, development of fibrosis mainly determines quality of life, as well as prognosis [3]. Thus, the level of fibrosis correlates with liver function and represents the major risk factor for development of hepatocellular carcinoma (HCC) [4]. Moreover, chronic portal hypertension due to liver fibrosis is the major cause of clinical complications, including hydropic decompensation, and bleeding events, as well as hepatic encephalopathy [3]. Consequently, liver cirrhosis is currently the 11th most common cause of death in the world [1] and the fourth most frequent cause of death in adults in central Europe [5,6].

**Figure 1:**
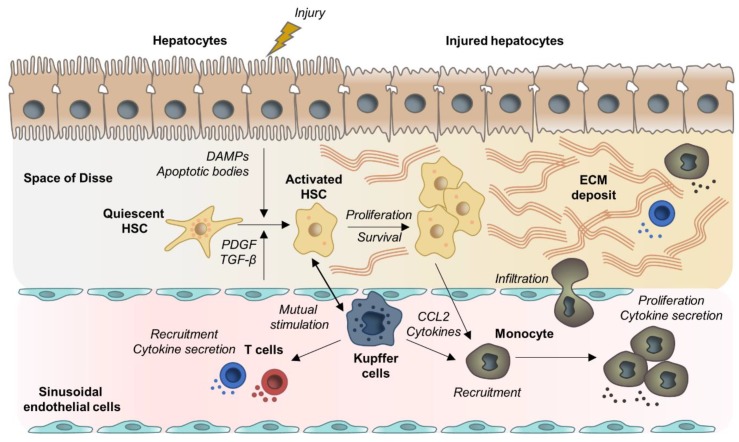
Examples for mechanistic concepts for liver fibrosis. Chronic hepatocyte injury causes release of damage-associated patterns (DAMPs) and apoptotic bodies that activate Hepatic stellate cells (HSCs) and recruit immune cells. Complex multidirectional interactions between activated HSCs and Kupffer cells, as well as innate immune cells promote trans-differentiation into proliferative and extracellular matrix (ECM) producing myofibroblasts. Abbreviations: PDGF: Platelet Derived Growth Factor; TGF-β: Transforming Growth Factor Beta; CCL2: chemokine (C-C motif) ligand 2.

Liver fibrosis is characterized by progressive accumulation of extracellular matrix (ECM), which destroys the physiological architecture of the liver [7]. Pathogenetically, toxic, metabolic, or viral diseases lead to damaged hepatocytes and infiltration of immune cells that activate trans-differentiation of Hepatic stellate cells (HSCs) into collagen-producing myofibroblasts [8,9]. Physiologically involved in tissue repair, upon short-term injury this process is balanced by counteracting anti-fibrotic mechanisms resulting in inactivation or apoptosis of myofibroblasts and scar resolution. In contrast, in chronic liver diseases an imbalance of pro-fibrogenic and anti-fibrogenic mechanisms causes persistent activation of proliferating, contractile, and migrating myofibroblasts that lead to excessive production of ECM [8,9]. The liver’s fate to either pass into an anti-fibrotic scar-dissolving stage or to proceed into an uninhibited fibrosis-promoting stage is hereby mainly regulated by non-parenchymal cells (NPCs), including Kupffer cells and other immune cells [10,11,12]. Thus, hepatocyte apoptosis and release of damage-associated patterns (DAMPs) by hepatocytes not only activate HSCs directly but also induce recruitment and activation of lymphocytes and macrophages that contribute to promotion of HSC trans-differentiation and myofibroblast activation by producing pro-inflammatory and pro-fibrogenic cytokines [13,14]. Distinct macrophage subpopulations on the other hand participate in fibrosis resolution due to expression of matrix-metalloproteinases (MMPs) [15,16]. On the molecular basis, a complex network of cytokine-induced signaling pathways orchestrate pro-fibrogenic cell interactions. In fact, Transforming Growth Factor Beta (TGF-β), Platelet Derived Growth Factor (PDGF), and the inflammasome (NLRP3)-Caspase1 pathway, as well as WNT/β-catenin signaling have been suggested to be key signaling pathways associated with HSC activation and fibrosis progression [17,18,19]. The general, etiology-independent cell interactions involved in fibrosis development are depicted in Figure 1.

One approach to prevent liver-related mortality is to prevent progression of fibrogenesis. Within the past years, several in-vitro and in-vivo models have been developed in order to address the unmet medical need of developing efficient and safe anti-fibrotic drugs [20,21,22,23]. However, despite increasing knowledge regarding the molecular mechanisms of liver fibrogenesis, an approved drug exist to treat liver fibrosis is still pending [24]. In this review we summarize recent advances in the understanding of cellular and molecular drivers of liver fibrogenesis in key etiologies of chronic liver disease. Moreover, anti-fibrotic strategies and agents in clinical development are discussed.

## 2. Mechanistic Concepts of Liver Fibrosis

### 2.1. Hepatocyte Cell Death and Apoptosis

Hepatocyte death is an important initial event in all liver disease etiologies. Dead hepatocytes release intracellular compounds termed DAMPs that send out danger signals to surrounding cells including HSCs and Kupffer cells and therefore play an important role in fibrosis development and inflammation. This family of molecules comprise nucleic acids, intracellular proteins, Adenosine Triphosphate (ATP), or mitochondrial or nucleic compounds such as High-Mobility Group Box-1 (HMGB1) [25]. DAMPs can be passively released by necrotic hepatocytes due to the disruption of plasma membrane [25,26]. HMGB1 is one of the most studied DAMPs in the context of liver disease. It is a DNA-binding non-histone nuclear protein ubiquitously expressed in eukaryotic cells. HMBG1 is highly released by necrotic hepatocytes as a danger pattern [26]. In addition, it can be secreted by stressed cells and contribute to immune responses and inflammation by interacting with the Toll Like Receptors (TLR) 4 and 9 [27,28,29,30]. Moreover, Li et al. recently provided evidence that HMGB1 directly activates HSCs by regulating HSCs autophagy in a model of HBV-related liver fibrosis progression [31]. Finally, it was recently demonstrated that HMGB1 plays an essential role in the recruitment of pro-inflammatory neutrophils to sites of necrotic injury in the liver [32].

In contrast, apoptosis generates low levels of DAMPs because the cell components are largely retained in apoptotic bodies [25,26]. However, a pro-fibrogenic response can be elicited by hepatocyte apoptosis through activation of the Fas death receptor [33,34]. Moreover, hepatocyte apoptosis induces the release of apoptotic bodies which can be phagocytosed by HSCs and Kupffer and induce a pro-fibrogenic response [35,36]. In addition, DNA from apoptotic hepatocytes triggers TLR9 activation on HSCs and collagen production [37].

Lipid overload in hepatocytes is one of the main drivers of hepatotoxicity, which accelerates the development of progressive inflammation, oxidative stress and fibrosis [38]. In the liver, lipids are mainly stored as triglycerides, an inert and non-cytotoxic form of lipid. Lipotoxicity is rather due to accumulation of toxic intermediates of triglyceride synthesis such as saturated Free Fatty Acids (FFAs) and their derivates, accumulation of free cholesterol or complex lipids as lysophosphatidylcholine and ceramides [39,40,41]. Accumulation of these lipids affect cellular function through different mechanism including oxidative and endoplasmic reticulum stress, mitochondrial dysfunction, and induction of apoptosis [38]. Accumulation of FFAs is one of the strongest apoptosis inducers in hepatocytes. This process is mainly mediated by the Tumor Necrosis Factor-Related Apoptosis-Inducing Ligand Receptor 2 (TRAIL-R2), also known as death receptor 5 TRAIL-R2 especially contributes to cell death caused by palmitic acid, which induces downstream activation of caspase 8 and executionary caspases 3 and 7 [42,43]. Moreover, FFA-induced lipo-apoptosis in hepatocytes stimulate the release of ATP, which stimulate migration of monocytes [44]. In addition to hepatocytes, NPCs are also impacted by the toxic lipid accumulation. FFAs accumulation in HSCs and Kupffer cells especially triggers TLR4 pathway activation, leading to c-Jun N-terminal Kinase (JNK) and NF-kB pathway activation, as well as secretion of pro-inflammatory and chemoattractant cytokines [38,45].

Dysregulation of hepatic cholesterol metabolism is also a key event leading to hepatocyte death. Free cholesterol causes hepatocyte apoptotic and necrotic death by activating JNK1 [46]. It has recently been shown that high concentration of free cholesterol in hepatocytes of NASH patients leads to cholesterol crystallization [47,48]. Dead hepatocytes containing cholesterol crystals induce the recruitment and aggregation of Kupffer cells in “crown-like structures”, which process dead cells and transform into activated foam cells [48]. Activation of Kupffer cells during this process contribute to HSCs activation through the release of pro-inflammatory cytokines. The group of Hibi et al. also demonstrated that accumulation of free cholesterol in HSCs directly exacerbate liver fibrosis [49,50].

### 2.2. HSC Activation and Myofibroblast Progenitor Cells

HSCs are the main myofibroblast progenitor cells and therefore key effectors of the fibrogenic response [51]. In normal liver, HSCs are quiescent, non-proliferative perisinusoidal cells, characterized by their star-like morphology and their high number of cytoplasmic lipid droplets [52]. Upon liver injury, HSCs become activated, and transdifferentiate from a quiescent phenotype into a proliferative and contractile myofibroblast phenotype [53]. During this process, activated HSCs progressively lose their star shaped morphology and their lipid droplets, while abundantly producing ECM components (including types I, III, and IV collagens, fibronectin, laminin, and proteoglycans) and pro-inflammatory mediators. In addition, activated cells express high levels of alpha Smooth Muscle Actin (α-SMA) and Tissue Inhibitor of Metalloproteinase 1 (TIMP1) which contribute to the changes from a adipocytic phenotype to a pro-fibrogenic and inflammatory phenotype [54,55].

Physiologically involved in tissue repair, following short-term injury myofibroblasts are rapidly cleared by apoptosis or inactivation [56]. However, under chronic injury, the persistent HSCs activation leads to disruption of the balance between ECM deposition and dissolution and triggers progressive liver fibrosis [51]. Moreover, in advanced fibrosis, the high number of activated HSCs and contractibility of the myofibroblasts promote the constriction of hepatic sinusoids, therefore affecting the blood flow and the nutrient exchange and participating in liver dysfunction [9].

Activation of HSCs consists of two major phases (i) the initiation, or pre-inflammatory stage, referring to the early changes in gene expression shortly after injury and (ii) the perpetuation, which corresponds to maintenance of an activated phenotype and fibrosis development [57]. The initiation stage is triggered by paracrine stimulation of HSCs through the products of injured hepatocytes, signals from the resident Kupffer cells and endothelial cells, as well as Reactive Oxygen Species (ROS) and lipid peroxides exposure [58]. Perpetuation results from the continuing effects of these stimuli. These signals induce enhanced proliferation, contractility, pro-inflammatory and chemoattractant mediator synthesis, and fibrogenesis/matrix degradation [57,58].

**Figure 2:**
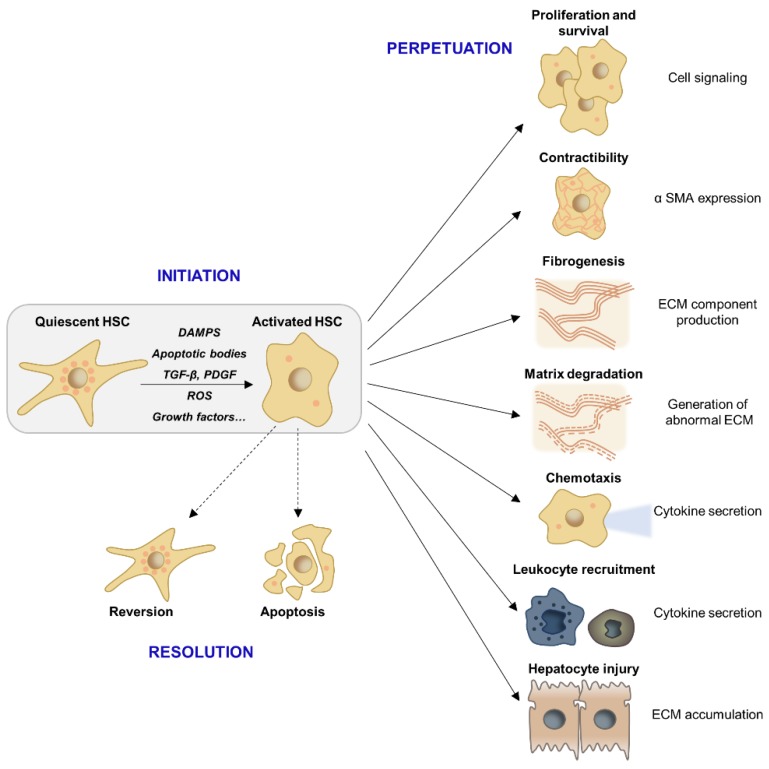
HSC activation and downstream pro-fibrogenic responses. Following the initial event of HSC activation, non-parenchymal cell directed pro- or anti-fibrogenic responses determine whether activated HSCs either transit into spontaneous resolution via reversion and apoptosis or pass into a perpetuated state that results in maintenance of a pro-inflammatory and pro-fibrogenic microenvironment as well as liver degrading ECM accumulation. Abbreviations: α-SMA: α-smooth muscle actin; DAMPS: Damage-associated molecular pattern; ECM: Extracellular matrix; HSC: hepatic stellate cells; PDGF: Platelet-derived growth factor; ROS: Reactive oxygen species; TGF-β: Transforming growth factor β.

The production of chemotactic and inflammatory substances induces the activation and the recruitment of other cellular effectors, including Kupffer cells, infiltrating immune cells, endothelial cells, and platelets, which reinforce the pro-fibrogenic environment and the maintenance of HSCs activation [53,59]. TGF-β and PDGF are the two major cytokines contributing to HSCs activation and proliferation. These two major pathways as well as further contributing mediators driving liver fibrosis (i.e., ROS) will be further discussed in this section [57,60]. All these signals lead to ECM accumulation in the extracellular space. Importantly, the matrix-degrading enzymes such as MMPs produced by HSCs and other pro-inflammatory effectors contribute to the replacement of normal ECM by an altered matrix. Indeed, the ECM remodeling involves changes in matrix stiffness, flexibility, and density related to the dysregulation of the components production [61] (Figure 2). Moreover, the ECM is not inert and can also store cytokines and growth factors secreted by the cellular effectors hereby further contributing to inflammation, fibrogenesis, hepatocyte proliferation, and carcinogenesis [53,61].

While activated HSCs are the predominant precursor of myofibroblasts in fibrotic liver (> 90% of collagen-producing cells), increasing evidence shows that myofibroblasts can also derive from portal fibroblasts [62,63], bone marrow [64,65], and some studies have suggested Epithelial-To-Mesenchymal Cell Transition (EMT) from hepatocytes or cholangiocytes [66]. However, the contribution of these cells in the development of liver fibrosis is still unclear and differ upon the different liver disease etiologies and stages. For example, the portal fibroblasts are mainly activated by cholestatic injuries and may initiate the periportal fibrosis [67,68]. Indeed, Iwaisako et al. reported that portal fibroblasts contribute to more than 70% of myofibroblasts upon biliary injury [68]. Regarding bone-marrow-derived myofibroblasts two potential sources have been described: fibrocytes and Mesenchymal Stem Cells (MSCs). The fibrocytes can differentiate into myofibroblasts and are recruited in the injured tissue over time, suggesting a role in advanced disease [62]. MSCs are multipotent progenitor that can differentiate into hepatic myofibroblasts [62,64] via mesothelial to mesenchymal transition upon chronic liver injury [69]. Nonetheless, their exact contribution to liver disease development is still controversial. On one hand, studies indicate their ability to differentiate into pro-fibrogenic myofibroblasts [70], on the other hand several studies demonstrated that injection of MSCs improves liver fibrosis/cirrhosis in mice and could be used as a novel therapeutic approach [71,72]. More studies are therefore needed to clarify the role of these cells. Finally, cholangiocytes and hepatocytes can develop a myofibroblast phenotype via EMT [66,73]. EMT is a reversible process by which epithelial cells lose their polarity and can differentiate into mesenchymal cells. TGF-β, the most potent pro-fibrogenic cytokine upregulated during liver fibrosis is known to be a strong inducer of EMT. However, some controversies remain. Indeed, lineage-tracing experiments have demonstrated that myofibroblasts found in experimental liver fibrosis do not originate from epithelial cells [74,75].

### 2.3. Liver Macrophages

Macrophages represent the largest NPC population in the liver and play a central role in liver inflammation and fibrosis. Hepatic macrophages comprise the liver resident Kupffer cells and monocyte-derived macrophages, originating from the bone-marrow [13]. Activation of Kupffer cells and recruitment of monocyte-derived macrophages are triggered by the release of DAMPs ROS production, anti-viral response but also by metabolic signaling induced by fat accumulation [76,77,78,79].

Macrophages can be classified into a wide spectrum of different phenotypes ranging from the classically activated pro-inflammatory macrophages (M1) to alternatively activated immunoregulatory macrophages (M2). These subclasses are induced by different regulators and exhibit distinct markers and functional activities. M1 are characterized by the expression of pro-inflammatory cytokines (Tumor Necrosis Factor alpha (TNF α), the interleukins (IL) IL6 and IL1β), whereas M2 express anti-inflammatory mediators (IL4, IL1) [80]. Hepatic macrophages exhibit a remarkable plasticity and can switch to different phenotypes in response to various stimuli of their microenvironment, sometimes expressing both markers of M1 and M2 differentiation. While being difficult to distinctly attribute this dichotomous classification to pro- or anti-fibrogenic actions [13,15], numerous studies indicate distinct subpopulations of macrophages to coexist in the liver and to contribute to different phases of fibrosis. Thus, Duffield et al. demonstrated that macrophage depletion in the early phase of injury decreases the inflammatory response and reduces scarring and the number of myofibroblasts. In contrast, macrophage depletion during recovery leads to a failure of ECM degradation and a less efficient repair [81].

In the early phase of the injury, the dominant macrophage populations are pro-inflammatory. The resident Kupffer cells rapidly secrete IL-1β, TNFα, chemokine (C-C motif) ligand 2 (CCL2), and CCL5 resulting in activation of HSCs and recruitment of other immune cells including monocyte-derived macrophages [15]. Monocytes infiltration into the liver is primarily controlled by C-C Motif Chemokine Receptor 2 (CCR2) and its ligand CCL2 and is a major contributor of fibrosis development [82,83]. Recruitment of pro-inflammatory cells is the principal driver of hepatic inflammation. Mutual stimulation of inflammatory cells and HSCs results in amplification and perpetuation of the pro-fibrogenic liver state (for a review, see [84]). Activated HSCs modulate immune cell recruitment via secretion of pro-inflammatory and chemoattractant molecules and by secreting ECM which constitutes a network for leukocytes migration and retention [85]. Activated macrophages secrete cytokines to stimulate HSCs, which in turn produce the macrophage colony-stimulating factor, IL6, and other cytokines to perpetuate the pro-fibrotic macrophage activity [85,86,87]. Moreover, Kupffer cell activation increases the activity of NF-kB in HSCs, which further promotes pro-inflammatory cytokine secretion [87]. Different studies also described a direct interaction of HSCs with immune cells through expression of adhesion molecules (ICAM-1, VCAM-1), resulting in mutual stimulation and amplification of the pro-fibrogenic response [88,89]. More recently, Lodyga et al. showed that cadherin-11 (CDH11) mediates adhesion of macrophages to myofibroblasts and establishes a pro-fibrotic niche of active TGF-β [90]. Another recent example of mutual stimulation between HSCs and Kupffer cells was reported by Cai et al.They demonstrated that CXCL6 plays an important role in liver fibrosis through stimulating the release of TGF-β by Kupffer cells via an EGFR-dependent pathway [91].

During progression of injury, macrophages exhibit intermediate phenotypes and switch to a most anti-inflammatory profile. These macrophages respond to IL10, IL4, and IL13 and secrete anti-inflammatory mediators such as IL-10 and TGF-β [92]. At this stage, some resident macrophages can have a wound healing phenotype characterized by the production of MMPs (i.e., MMP9, MMP12, MMP1), which are involved in matrix degradation and resolution of fibrosis [11,16]. During late-stage injury, the dominant macrophage population is anti-inflammatory due to the abundance of the TGF-β in the fibrotic environment [93]. These macrophages progressively switch to an immunosuppressive phenotype, allowing immune evasion and tumor promotion. Indeed, they produce immunosuppressive mediators such as IL10 and express cell surface receptors like programmed cell death 1 ligand 1 (PD-L1) and the receptor sialic-acid-binding Ig-like lectin 10 that play major roles in suppressing the immune system [94,95,96]. Therefore, TGF-β provides a link between liver fibrosis and immune responses.

The controlled inflammation and macrophage activation at the different stage of liver injury is an essential feature to control fibrosis development. However, due to the remarkable plasticity of macrophages, translation of this concept into clinical application is challenging. The precise contribution of each macrophage population needs to be fully dissected in the future.

### 2.4. Lymphocytes

While the role of myofibroblasts and macrophages in fibrogenesis is well described, the role of adaptive immune cells is less defined. Nevertheless, the importance of lymphocytes in fibrogenesis is evidenced by in vivo studies showing that inhibition of lymphocyte recruitment in the liver induces a decrease in the fibrogenic responses [97,98,99,100].

As described above, chronic liver injury results in the production of pro-inflammatory mediators and the infiltration of leukocytes, including lymphocytes, in the sub-endothelial space. The recruitment of lymphocytes from the circulation is further triggered by interactions with endothelial cells, a process regulated by several chemokines. Importantly, lymphocytes can interact with ECM components and endothelial cells though cell surface integrins, which contribute to cell activation and differentiation as well as fibrogenic responses [101,102]. After migration through the endothelium by a complex mechanism, lymphocytes are recruited at the injury site by chemoattractant molecules [88]. It has been shown that CXCR3 activation by its ligands, including CXCL9, CXCL10, and CXCL11 produced by HSCs and endothelial cells, promotes lymphocytes trans-endothelial migration [103]. Myofibroblasts also secrete cytokines promoting lymphocyte migration, including IL-6, hepatocyte growth factor and TGF-β [97].

CD4+ T cell responses have been shown to be critical for fibrosis development. Polarization of CD4+ T cells in distinct T-helper (TH) lineages is critical for defining cell properties and cytokine production. The TH2-polarized T cells are directly involved in fibrosis development by stimulating pro-fibrogenic gene expression in myofibroblasts (pro-collagen I and III, MMP2, MMP9, and TIMPs) and the synthesis of immunoregulatory mediators in macrophages (IL10, TGF-β) [14]. These processes are mainly driven by IL4 and IL13 [14,104]. IL17-producing CD4+ T cells and regulatory T cells (Tregs) have also been identified as effectors of liver fibrogenesis. It was observed that IL-17 expression is upregulated in fibrotic liver tissue and promotes pro-inflammatory cytokine expression, neutrophil influx, liver injury, and fibrosis [105,106]. Similarly, an increase of the Treg population was observed in patients with advanced fibrosis, which may promote fibrosis through secretion of IL8 [107,108]. The role of B-lymphocytes and CD8+ T cells in liver fibrosis is less well understood. It is possible that these cells could promote fibrosis by secreting pro-fibrotic cytokines or by amplifying tissue injury [88,97,109].

### 2.5. Gut Dysbiosis

Numerous studies suggest a key role of gut dysbiosis in the progression of liver fibrosis. The term liver-gut axis describes the close bidirectional interaction between the gut and its microbiota with the liver. Thus, 75% of the portal vein blood derives from the gut and transports intestinal products to the liver. The liver in turn secretes bile and antibodies into the gut [110]. The interface between the liver and the gut microbiota is shaped by the mucosal barrier, consisting of the gut epithelial barrier and the gut vascular barrier. The integrity of this intestinal mucus barrier and the physiological composition of the intestinal microbiome are critical for maintaining homeostasis of the liver-gut axis [111]. Metabolic toxins, especially alcohol abuse or high fat/low fiber diet in NAFLD have been described to disrupt intestinal homeostasis by increasing intestinal permeability and altering microbiota [112,113]. Consequentially, the relative overgrowth of potentially pathogenic bacteria not only drives hepatic inflammatory immune responses and HSC activation due to portal delivery of pathogen-associated molecular patterns (PAMPs, as lipopolysaccharides, peptidoglycans, and flagellin), the altered microbiome also results in intestinal deconjugation of bile acids and therefore production of so-called secondary bile acids that suppress Farnesoid-X Receptor (FXR) signaling [111]. FXR is a nuclear receptor activated by bile acids that regulates bile acid, lipid, and glucose metabolism [114,115]. Intestinal FXR signaling physiologically exert protective effects on intestinal epithelial barrier properties [116] and accelerates gut vascular barrier repair [113]. Intestinal accumulation of secondary FXR- suppressing bile acids in chronic liver disease therefore promotes disruption of the intestinal barrier.

Independent of the underlying etiology and presence of the causal toxin, fiver fibrosis itself is typically accompanied by gut dysbiosis [117,118]. These etiology-independent alterations in the gut microbiome [117,119] are due to reduced small bowel motility e.g., in the context of ascites [119,120,121] and compromised intestinal immunity [122]. Moreover, highlighting the reciprocal interaction of bile acids and the gut microbiome, reduced excretion of primary bile acids in liver fibrosis with compromised liver function directly affects composition of the gut microbiome [119,121]. Typical features of gut dysbiosis in liver cirrhosis are reduced diversity and relative overgrowth of potentially pathogenic bacteria as *Enterococcaceae* and *Enterobacteriacae* or bacteria of buccal origin [118]. Together with the typical severely compromised gut barrier, gut dysbiosis promotes cirrhosis inflammatory state due to hepatic accumulation of PAMPs and toxic bacteria products [123] and correlates with liver disease progression [124,125]. Nevertheless, abundance of pathogenic taxa associates with risk of decompensation in patients with liver cirrhosis and enteral bacterial translocation is involved in outcome-determining complications as spontaneous bacterial peritonitis and hepatoencephalopathy [124,126].

### 2.6. Molecular Signaling Pathways Involved in Liver Fibrogenesis

#### 2.6.1. PDGF Signaling

PDGF is a growth factor promoting HSCs division and proliferation. Four different PDGF subunits, termed PDGF-A, -B, -C, and -D, were identified and can produce five different polymers (PDGF-AA, -BB, -AB, -CC, and -DD), via a disulfide bond linkage, which have different functions [127]. PDGF-AA mainly controls cell proliferation and chemotaxis, while PDGFR-AB and -BB promote collagen synthesis [17]. Moreover, several studies demonstrated that the subunit PDGF-B is the most potent factor associated with early HSCs activation. Indeed, PDGF-B expression is transiently increased during the early stage of activation. In contrast, PDGF-C and -D levels are increased during the trans-differentiation and persist upon the perpetuation, suggesting a role of these subunits in the late phase of fibrogenesis [128,129,130].

Under healthy conditions, PDGF is produced by platelets. During liver injury, Kupffer cells mediate intrahepatic recruitment of platelets [59]. Moreover, PDGF can also be expressed by Kupffer cells, endothelial cells, and activated HSCs. Finally, PDGF receptor (PDGFR) is expressed at the membrane of HSCs and can therefore stimulate HSCs activation through autocrine mechanism [131,132].

The binding of PDGFs on their corresponding receptors induces receptor dimerization and phosphorylation which in turn phosphorylate tyrosine residues on different intracellular substrates. Stimulation of PDGFR triggers activation of several signaling pathways including the Ras/Raf system, the phospholipase Cγ (PLCγ), the phosphatidylinositol 3-kinase (PI3K)/Akt pathway, and the JAK/signal transducer and activator of transcription (STAT) pathway [17]. These downstream elements then regulate the expression levels of pro-fibrotic target genes such as type I collagen (COL1A1), metalloproteinase inhibitors (TIMPs), and MMPs but also the apoptosis regulator Bcl 2, resulting in cell proliferation and survival [17].

#### 2.6.2. TGF-β Signaling

In cooperation with PDGF, the TGF-β signaling is considered as one of the most important pathways driving HSC activation and fibrogenesis [133]. The TGF-β family comprises 33 members. While TGF-β2 plays an important role in biliary fibrogenesis, TGF-β1 is the most widely investigated isoform in liver fibrogenesis [134]. TGF-β is synthetized as a latent precursor by a variety of cells including endothelial cells, macrophages, and hepatocytes. Moreover, platelets were recently identified as an important source of TGF-β in the liver [135]. The inactive TGF-β molecules bind to the latency associated protein (LAP) and accumulate in the ECM and must be cleaved by specific proteases to become active. Endothelial cells participate in the conversion of TGF-β from the latent to the active form. Moreover, interactions with transmembrane integrins are considered as the principal activating mechanism for latent TGF-β [136]. The active form binds to and activates the TGF-β type II receptor (TβRII), which recruits the TGF-β type I receptor (TβRI). The downstream canonical signaling of TGF-β1 converges on SMAD proteins.

The SMAD protein family can be classified into three groups based on their functions. The receptor-regulated SMADs (R-SMADs) include SMAD1, SMAD2, SMAD3, SMAD5, and SMAD8. The inhibitory SMADs include SMAD6 and SMAD7. SMAD4 is the only member of the third category, named common SMAD. R-SMADs are activated by phosphorylation at their C-terminus, i.e., pSMAD2 and pSMAD3, and form a complex with SMAD4, which translocates into the nucleus to regulate gene expression. SMAD3 is crucial for inducing HSCs activation and pro-fibrogenic gene transcription such as α-SMA or COL1A1 [116]. Of note, activation of the SMAD3-dependent TGF-β signaling in hepatocytes was also demonstrated to contribute to fibrosis development, especially in NASH, by inducing hepatocyte death and lipid accumulation [137]. In contrast to SMAD3, SMAD2 has no DNA binding capacity and is described as an anti-fibrotic molecule. The underlying mechanism could involve the ability of SMAD2 to induce TRAIL-mediated HSC apoptosis [138]. Moreover, SMAD6 and SMAD7, which negatively regulate TGF-β signaling, are considered as anti-fibrotic factors [139,140]. As a proof of concept, the group of Mertens showed that overexpression of SMAD7 in transgenic mice interferes with liver fibrosis progression and liver damage [141].

The canonical pathway in which SMAD3 is phosphorylated at its C-terminus (pSMAD3C) is described as the main fibrogenic pathway [60,142]. However, a non-canonical and pro-fibrogenic pathway was recently described, in which PDGF activates JNK that phosphorylates SMAD3 in its linker domain (pSMAD3L). PSMAD3L is able to rapidly translocate into the nucleus to stimulate HSC proliferation and induces a pro-fibrogenic response [143,144]. This non-canonical pathway is therefore crucial for the induction of ECM production and is now considered as an attractive therapeutic target [143]. Other studies have also identified TGF-β non-canonical pathways such as mitogen-activated protein kinase (MAPK), mammalian target of rapamycin (mTOR), PI3K/Akt, JAK1/STAT3, and Rho GTPase pathways. Both the canonical and the non-canonical pathways contribute to HSCs activation but also to macrophages activation and polarization [136]. TGF-β production is also associated with the activation of the connective tissue growth factor (CTGF) in HSCs and hepatocytes, a mitogenic factor playing an important role in liver fibrosis development [60]. Finally, it was shown that ROS can act as inducer or effector of the TGF-β signaling and therefore generate a vicious cycle for fibrosis [145]. Moreover, high levels of TGF-β induce a massive hepatocyte cell death, contributing to chronic liver damage [88].

#### 2.6.3. Oxidative Stress

Oxidative stress (OS) is a key process driving liver damage and initiation liver fibrosis. It corresponds to an altered balance between cellular pro-oxidant and antioxidant factors, which results in ROS and reactive nitrogen species (RNS) production. ROS constitute a family of pro-fibrotic mediators including superoxides, hydrogen peroxide (H_2_O_2_), and hydroxyl radicals [146]. They are generated during normal cellular metabolism and in particular during oxidative phosphorylation and lipid peroxidation in hepatocytes, HSCs, and macrophages. At low levels, ROS can serve as secondary messengers to activate different cellular responses [147]. However, at high levels, they provoke disruption of cellular lipids, proteins and DNA and lead to hepatocyte necrosis and apoptosis. Moreover, ROS stimulate pro-inflammatory and pro-fibrogenic factor production by activated HSCs, Kupffer cells, and other pro-inflammatory cells [77,148]. ROS production is exacerbated by ethanol, FFA accumulation, iron deposit, and chronic viral infection [146,149,150].

The NADPH oxidases (NOXs) are a major source of ROS in the liver and mediate fibrogenic responses induced by angiotensin II, PDGF, and TGF-β in HSCs and macrophages [151,152]. Zhan et al. notably showed that phagocytosis of apoptotic bodies by HSCs following hepatocyte death results in NOX activation and collagen production [35]. Other studies demonstrated that the TGF-β-SMAD3 pathway increases NOX1 and NOX4 expression in HSCs, which correlates with the degree of fibrosis [153,154,155]. ROS signaling also regulates the expression and the activity of the transcription factor NF-κB. NF-κB has a key role in the regulation of cell death, inflammation, and wound healing and is therefore an important modulator of liver fibrosis progression [156]. Indeed, several studies have shown that inhibition of NF-κB activity protects from hepatic fibrosis in-vivo [157]. Moreover, in contrast to quiescent HSCs where NF-κB activity is suppressed, myofibroblasts display a high NF-κB activity, suggesting that NF-κB activity is linked with HSC proliferation [156]. In line with this observation, it was demonstrated that Kupffer cell activation increases the activity of NF-kB in HSCs, which in turn promotes pro-inflammatory cytokine secretion [87,156].

Over the last few years, epigenetic regulation of fibrosis progression has emerged as another process which orchestrates several aspects of the fibrogenic response in the liver (for a review, see [158]). Important epigenetic changes are induced by ROS in the HSCs, including chromatin remodeling by histone modification, DNA methylation and gene silencing by microRNAs (miRs) [158]. In-vitro and in-vivo approaches have demonstrated that HSCs show a global demethylation of fibrogenic genes during transdifferentiation into myofibroblasts, which is associated with liver fibrosis development [159,160,161].

#### 2.6.4. The Inflammasome (NLRP3)-Caspase1 Pathway

Hepatic inflammation is a pan-etiology driver of hepatic damage and liver fibrosis. Inflammasomes are intracellular multiprotein complexes expressed in hepatocytes and NPCs including HSCs and Kupffer cells [162]. From the various inflammasomes, the NOD-like receptor (NLR) NLRP3 inflammasome is the best characterized. It has been shown to play a crucial role in the progression of NAFLD to NASH [163,164]. NLRP3 inflammasome consists of an intracellular multiprotein complex that activates caspase 1 by cleavage, which further cleaves pro-IL1β and pro-IL18 into mature forms. IL1β and IL18 are important mediators of the innate inflammatory response which initiate and perpetuate an abnormal wound-healing response and facilitate the progression of hepatic fibrosis.

Even if NLRP3 inflammasome activation in different cell types has not been completely elucidated, several evidences demonstrated that accumulation of toxic lipids and DAMPs- and PAMPS-mediated TLR signaling activates NLRP3 inflammasome [45,163,165]. It was notably demonstrated that TLR2 and palmitic acid cooperatively activate NLRP3 inflammasome in Kupffer cells and promote HSCs activation through pro-inflammatory cytokine secretion [166]. Moreover, it was speculated that phagocytosis of cholesterol crystals from hepatocytes can activate NLRP3 inflammasome in macrophages and may contribute to inflammation and fibrosis in NASH [47]. Finally, it was shown that activation of NLRP3 in hepatocytes results in pyroptosis, a form of programmed cell death involving caspase 1, liver inflammation, and fibrosis [167]. Therefore, blockade of NLRP3 pathway emerges as a novel therapeutic target to reduce liver inflammation and fibrosis in NASH [168].

#### 2.6.5. Wnt/β-Catenin Signaling

Physiologically, the Wnt/β-catenin pathway is necessary for organ development. However, Wnt/β-catenin signaling has also been associated with the development of fibrosis in different organs, including the liver [19]. β-catenin is a protein which acts as both adhesion molecule and transcription factor. Its expression is regulated by the Wnt protein. When the pathway is inactive, β-catenin level in the cytoplasm is regulated by a destruction complex which includes the glycogen synthase kinase 3β and casein kinase 1α. In contrast, when the pathway is active, Wnt binds the receptor Frizzled and the low-density lipoprotein-receptor-related protein 5/6 to form a complex, which inhibits β-catenin degradation. β-catenin in turn translocates in the nucleus to activate target genes transcription. However, β-catenin must recruit coactivators to be fully active, such as p300 or the cyclic AMP response element-binding protein-binding protein (CBP) [19]. During liver injury, the Wnt signaling is upregulated in the HSCs compared to quiescent cells and contribute to the pro-fibrogenic response by promoting α-SMA expression and collagen deposition [169].

## 3. Disease-Related Pro-fibrogenic Mechanisms in Chronic Liver Diseases

### 3.1. Chronic Hepatitis C

Chronic hepatitis C affects around 70 million people worldwide and still represents a leading cause of HCC and liver transplantation [170]. In most cases, infection by the hepatitis C virus (HCV) does not resolve spontaneously. Thus, approximately 80% of infected patients become chronic carriers and 20–30% develop liver cirrhosis within 25–30 years [171]. Chronic hepatitis C can now efficiently be cured by direct acting antivirals (for review see [172]). Chronic HCV infection induces hepatocyte cell death, that leads to release of DAMPs that can directly activate HSCs [31,35,77]. However, chronic inflammation due to antiviral immune response is still regarded as the most important driver of myofibroblast activation and ECM production in HCV infected patients [173]. Thus, immune response to HCV infection results in enhanced secretion of multiple growth factors, inflammatory cytokines, and chemokines by Kupffer cells and lymphocytes [174,175]. Moreover, HCV replicating hepatocytes have been shown to secrete pro-fibrogenic cytokines [176].

HCV viral proteins have also been shown to directly modulate signaling and metabolic pathways implicated in fibrogenesis. Thus, several studies indicate activation of HSCs into myofibroblasts by the HCV core protein, as well as non-structural HCV proteins. In fact, HCV core protein activates HSC proliferation in an Ras/ERK and PI3K/AKT dependent manner. The non-structural NS3 and NS5 proteins on the other hand induce inflammatory signaling pathways, including NF-κB [177]. Moreover, hepatocyte expression of HCV core protein is associated with decreased intracellular and mitochondrial glutathione levels, an important antioxidant [178,179]. Further promoting oxidative stress, the HCV protein NS3 can directly activate NOX2 in Kupffer cells and T cells [149,180]. The HCV envelope protein E2 on the other hand has been shown to bind to CD81 on HSC and actives MMP2, which have been hypothesized to promote inflammatory infiltration and enhanced parenchymal damage due to degradation of normal hepatic ECM [181]. Finally, human myofibroblasts have been reported to express HCV host factors and to be permissive to HCV. Increased proliferation and collagen production in these cells indicates further potential direct pro-fibrogenic effects of HCV on these fibrosis-driving cell population [182].

While cure of HCV infection has been shown to reduce liver disease complications and HCC risk, a significant risk to develop HCC persists in advanced fibrosis [183,184]. Several studies have shown that chronic HCV infection results in persistent epigenetic and transcriptional changes associated with the stage of fibrosis and HCC risk [185,186], suggesting that viral cure only partially eliminates the virus-induced pro-fibrogenic and carcinogenic drivers particularly in advanced fibrosis [187].

### 3.2. Chronic Hepatitis B

Despite the presence of an efficient vaccine, chronic HBV infection still affects currently approximately 260 million people, mostly in Africa and Asia [188]. While horizontal transmission of adults often results in self-limiting acute infection, vertical transmission mostly leads to chronic infection [189,190]. Currently available therapeutic therapies for chronically infected patients include interferon-based therapies and several nucleos(t)ide analogues. While nucleos(t)ide analogues rarely result in viral cure, suppression of viral replication slows down disease progression, that can eventually end in liver cirrhosis and HCC [191]. As in chronic hepatitis C, chronic hepatitis B triggers HSCs activation via DAMPs release and the host antiviral immune response leading to chronic inflammation [31,35,76,77,192]. However, in contrast to HCV, the direct involvement of HBV infection in HSC activation remains less defined. A recent study showed that the HBV encoded x protein (HBx) induces overexpression of the special AT-rich binding protein 1 (SATB1) in hepatocytes, which in turn promotes the activation and proliferation of HSCs through the secretion of CTGF and PDGF [193]. Moreover, Liu et al. observed that HBV can transiently infect and replicate in cultured HSCs in-vitro and that production of HBV S protein (HBs) affects their proliferation and expression of collagen type I [194]. Moreover, a direct activation of Tregs by HBx was observed in HBV-infected patients [105,106]. While pharmacological control of HBV infection markedly reduces liver disease progression and HCC risk, the absence of effective curative therapies still poses a challenge for the long-term management of patients [195].

### 3.3. Alcoholic Liver Disease

Alcoholic liver disease is a major cause of liver fibrosis world-wide. Chronic alcohol intake activates pro-fibrogenic mechanisms: the metabolization of alcohol in hepatocytes to acetaldehyde causes release of ROS, that can activate HSCs in a paracrine way [196]. Moreover, the ethanol metabolite acetaldehyde itself is fibrogenic and induces secretion of TGF-β [197]. Furthermore, both collagen type 1 genes have acetaldehyde-responsive elements that allow acetaldehyde-induced collagen expression in HSCs within hours [197,198]. Several studies further indicate alcohol-induced apoptosis of hepatocytes as a mechanism of liver fibrosis. Thus, hepatocyte apoptosis increases upon alcoholic liver injury [199], which not only induces production of chemokines and pro-inflammatory cytokines that activate HSCs [200] but also induces phagocytosis of the apoptotic bodies by Kupffer cells, that become pro-fibrogenic and release HSC activating cytokines as TNFα and TGF-β [36,201,202,203]. Finally, chronic alcoholic intake has been correlated with suppression of innate immunity [204,205,206]. Innate cytokines [207], natural killer (NK) cells [208], and macrophages [209] have been reported to inhibit liver fibrosis by clearance or inactivation of HSCs and therefore may underlie decompensation of the physiological balance of pro- and anti-fibrogenic mechanisms in chronic ASH.

### 3.4. Non-Alcoholic Liver Disease

NAFLD represents the fastest growing etiology of chronic liver disease and currently affects 15-30% of the global adult population [210] with expected further exponential increase within the next years [211]. NASH describes currently the inflammatory form of NAFLD characterized by disease progression and increased HCC risk. For many years, diagnosis of NASH required the exclusion of other potential triggers of chronic liver disease as alcohol abuse or viral infection. However, due to the variety of etiologies and pathologies there is overlap. Recently, another term “metabolic associated fatty liver disease (MAFLD)” has been suggested as a more appropriate and defining nomenclature for the heterogeneous population of patients with this disease. By avoiding the description “non-alcoholic”, this new terminology is supposed to address the high prevalence of co-existing toxic (e.g., alcohol) or viral contributors that do not exclude the affiliation to a metabolic liver disease [212]. Instead, thorough patient stratification according to present risk factors and chronic liver disease contributors should be performed to allow preventive and therapeutic recommendations that address the underlying disease in its whole complexity [212].

HSCs activation by oxidative stress and inflammation plays a leading role in NASH disease progression and fibrosis development [213]. Accelerated by insulin resistance accumulating metabolites of saturated fatty acids cause lipotoxicity that damages hepatocytes and results in oxidative stress [214,215]. Hepatocyte released DAMPs activate Kupffer cells via TLR and hereby create a pro-inflammatory microenvironment that promotes a HSC activating fibrogenic adaptive immune response [216]. Moreover, it has been shown that the high levels of oxidative stress in NASH hamper the physiologic regenerative proliferation of mature hepatocytes [217] and triggers recruitment of hepatic progenitor cells. These cells form the so called ductular reaction at the interface of hepatocytes and the biliary tree and are able to differentiate into both hepatocytes and cholangiocytes [218]. Of note, pro-fibrogenic cytokines, including TGF-β have been shown to be released by the ductular reaction [219]. Moreover, it has been shown that cholangiocytes can transform into collagen-producing myofibroblasts by EMT [220]. Further highlighting the potential role of HPC expansion/ductular reaction in NASH associated fibrosis progression, portal fibrosis that represents a key feature in progressive NASH livers correlates with the extent of ductular reactions and the degree of fibrosis [221]. However, demonstrating ECM accumulation and myofibroblast activation prior to PLC expansion in a murine mouse model of NASH, Van Hul et al. elegantly indicated LPC expansion to be only part of the complex pathogenesis of fibrosis in NASH, that is further depending on the inflammatory microenvironment [222]. While there is a large pipeline of compounds for treatment of NASH, there are currently no approved therapies [223].

## 4. Resolution of Liver Fibrosis

Progression into liver fibrosis and cirrhosis account for high morbidity and mortality in patients with chronic liver diseases, causing substantial economic burden. Patients with compensated liver cirrhosis have a 2–7% risk for hepatic decompensation and 1–7% risk of HCC development per year [224]. In NASH patients, fibrosis is the only histological feature that independently correlates with clinical outcomes [225,226,227]. Emphasizing the urgent need for efficient anti-fibrotic drugs, liver cirrhosis is currently the 11th most frequent cause of death worldwide [1].

Removal of the main inducer of chronic inflammation have been shown to be able to induce regression of advanced liver fibrosis (up to Metavir stages 3 and 4) due to chronic HBV and HCV infection [228,229,230]. However, approximately 15% of patient with chronic viral liver infection do not show any reversal in liver fibrosis despite sustained viral response [228,229]. In metabolic liver disease, lifestyle changes and bariatric surgery can induce regression of histological fibrosis [231], however, licensed therapeutic compounds for NASH are absent. Finally, spontaneous resolution after removal or treatment of the trigger of chronic inflammation occurs slowly and may not prevent life-threatening complications. Thus, besides causal therapies of underlying pathologies of chronic liver disease, anti-fibrotic strategies are needed to inhibit trigger-dissociated progression of liver fibrosis and to accelerate fibrosis resolution.

### 4.1. Molecular Mechanisms of Fibrosis Regression

Fibrosis regression is associated with inactivation or apoptosis of HSCs and myofibroblasts [56,232]. Thus, whereas increased cell death in hepatocytes contributes to fibrogenesis, cell death in HSCs is an important mechanism for the resolution of liver fibrosis. Indeed, TRAIL-mediated HSCs apoptosis is associated with an improvement of liver fibrosis [233,234,235]. Dissolution of the fibrotic scar is mainly mediated by macrophages that secrete the matrix-degrading enzymes collagenase and MMPs [10,16]. Macrophages associated with the resolution of hepatic fibrosis have been termed scar-associated macrophages (SAMs) and exhibit a phenotype outside the M1/M2 classification [16]. Thus, while pro-fibrotic macrophages have been characterized by a high expression of Ly-6C or Gr1 [16], CD11b^neg^ macrophages with low expression of Ly-6C are associated with MMPs production and fibrosis resolution [236,237]. Using single-cell RNA-Seq of patient-derived liver tissue, Ramachandran et al. elegantly demonstrated that distinct macrophage subpopulations inhabit the fibrotic niche [238]. Moreover, they identified a novel scar-associated TREM2+ CD9+ subpopulation of macrophages with a hybrid phenotype, which expands in liver fibrosis and is pro-fibrogenic.

In addition to macrophages, NK cells exhibit an anti-fibrotic activity by mediating HSCs apoptosis through the production of interferon gamma (IFNγ) [239,240,241,242,243]. Moreover, activation of NK cells and their cytolytic activity are important to control premalignant cell growth in fibrotic environment [244].

### 4.2. Candidate Targets and Pathways for Therapeutic Intervention

Generally, anti-fibrotic therapies can be divided into agents that mediate its anti-fibrotic effects by i) hepatocyte protection, ii) inhibition of HSC activation and fibrotic scar evolution, or iii) immune modulation (for recent reviews see [24] and [245]). Moreover, several phytodrugs have been characterized to potentially exert multidimensional protective effects on liver fibrosis progression [246,247].

However, despite numerous preclinical and clinical trials, to date, no Food and Drug Administration (FDA)-approved anti-fibrotic drugs exist and the only available curative treatment option for patient with advanced liver cirrhosis is liver transplantation [248]. Examples of anti-fibrotics, that are currently in clinical trial are reviewed in the following and further summarized in Table 1, Table 2 and Table 3.

#### 4.2.1. Hepatic Protection via Inhibition of Apoptosis

Hepatocyte cell death by apoptosis is a major trigger of inflammation and HSC activation in the evolution of liver fibrogenesis in all etiologies [249,250]. Accordingly, inhibition of hepatocyte apoptosis decreased HSC activation in animal models of liver fibrosis [251,252]. Following a promising pre-clinical study in a carbon tetrachloride (CCl_4_)-based liver fibrosis rat model [253], just recently two randomized placebo-controlled trials investigated the pan-caspase inhibitor Emricasan in NASH patients with F1-F3 fibrosis [254] or cirrhosis with severe portal hypertension [255]. Garcia-Tsao et al. reported small reductive effects on hepatic venous pressure gradient (HVPG) in cirrhotic NASH patients [255]. No effects were seen in patients with acutely decompensated cirrhosis [256]. In contrast, 72 week administration of Emricasan in patients with NASH-associated F1-F3 fibrosis did not improve liver inflammation or fibrosis but rather tended to worse hepatocyte-ballooning, potentially due to activation of other mechanisms of cell death and necrosis [254]. Results from a recently completed clinical trial of Emricasan in the setting of post-transplant HCV-induced fibrosis after SVR are awaited 2020 (NCT02138253).

Another approach to reduce liver injury associated hepatocyte cell death is to inhibit stress signals. Apoptosis signal-regulating kinase (ASK1) belongs to the MAPK pathways and is involved in hepatic apoptosis, inflammation and fibrosis [257,258,259]. The selective ASK1 inhibitor Selonsertib improved fibrosis in a murine NASH model [257]. In a multicenter phase II clinical trial, 24 week treatment of patients with NASH F2-3 improved histological degree of fibrosis [260]. However, considering frequently reported improvement of fibrosis due to enforced patient’s compliance and therapeutic monitoring, the absent inclusion of a placebo-control group in this study substantiates the need for further confirmatory studies. Phase 3 clinical trials in patients with NASH associated F3 (NCT03053050) and F4 fibrosis (NCT03053063) have just been completed and results are awaited to be published in 2020.

#### 4.2.2. Hepatic Protection via Reduction of Oxidative Stress

Oxidative Stress is one of the major drivers in liver fibrosis progression, especially in NASH [261]. Consequently, several strategies to reduce oxidative stress have been developed and investigated in terms of anti-fibrotic potency [262,263,264,265]. NOXs are membrane-bound enzyme complexes that catalyze the reduction of NADH, hereby producing superoxide radicals. NOX 1, 2, and 4 exert key roles in the activation of HSCs during liver fibrogenesis [155,266] and NOX4 is involved in hepatocyte apoptosis [155]. GKT137831, a dual NOX1/4 inhibitor suppressed ROS production in HSCs in-vitro and in-vivo and significantly attenuated liver fibrogenesis in CCl_4_ and bile duct ligation based mouse models of liver fibrosis [267]. According to a first interim analysis of a phase 2 clinical trial in patients with primary biliary cholangitis, GKT137831 showed significant effects on serological cholestasis parameters. Publication of effects on additional endpoints, including fibrosis after a treatment duration of 24 weeks are expected to be published soon (NCT03226067).

#### 4.2.3. Hepatic Protection via Restoration of Gut Microbiome

Considering the pathophysiological implication of gut dysbiosis in chronic liver disease progression and fibrogenesis, numerous studies investigated the potential of probiotics, prebiotics, and fecal microbiota transplantation for anti-fibrotic therapy [268]. Probiotics are living micro-organisms and prebiotics are indigestible food ingredients that are supposed to improve or restore the gut microflora. Confirming the pathological relevance of gut dysbiosis in chronic liver diseases, prebiotics and probiotics have shown protective effects on steatosis and liver inflammation in animal models of chronic liver injury [269,270,271,272]. In line with the pre-clinical data, VSL#3, the most studied probiotic formulation, showed potential anti-inflammatory and insulin-sensitizing effects according to a meta-analysis in NASH/NAFLD patients [273]. Recently, Bajaj et al. reported association of a diet rich in cereals, fermented milk, vegetables, and coffee/tea, with microbial diversity and lower risk of hospitalization in cirrhotic patients [125]. However, evidence for systematical clinical application of pro- and prebiotics is still lacking due to limitations of clinical studies in sample size, placebo-control and precise information regarding patients’ diet and lifestyle [268].

Fecal microbiota transplantation (FMT) describes the transfer of a fecal suspension from a healthy donor into the intestine of a patient. Interestingly, FMT reduced liver injury in a mouse model of alcohol-induced chronic liver disease [269]. Moreover, FMT was superior to probiotics in prevention of hepatic encephalopathy due to protective effects on intestinal mucosal barrier function [274]. Few small clinical trials further indicated potential protective effects of FMT on chronic liver disease progression. Thus, Philips et al. reported single FMT in patients with severe ASH to reduce hepatic inflammation and improve survival during one year of follow-up [275]. Moreover, a randomized clinical trial with 20 patients with cirrhosis and recurrent hepatic encephalopathy revealed improved cognition and reduced hospitalizations following FMT compared to standard care [276]. However, lethal *Escherichia coli* bacteremia have been reported in patients that have undergone FMT [277]. Thus, further studies are needed to evaluate the potential and especially safety profile of FMT in chronic liver disease patients that are at high risk of bacteremia due to bacterial translocation.

#### 4.2.4. Hepatic Protection via Lipid-Lowering Agents

Statins are widely used lipid-lowering agents that decrease serum cholesterol levels by inhibition of the activity of 3-hydroxy-3-methylglutaryl co-enzyme a reductase [278]. Considering its lipid lowering properties, several studies addressed the consequential hypothesis of statins to decrease experimental liver steatosis with controversial results [279,280,281]. However, recent evidence for independent pleiotropic effects of statins on chronic liver diseases have led to increasing interest among hepatologists (for a recent review see [282]). Thus, several studies on animal models of liver fibrosis reported statins to decrease oxidative stress, hepatic inflammation, and fibrogenesis [283,284,285]. Moreover, retrospective analyses of patients with chronic liver diseases and hypercholesterinemia-indicated statin-use revealed association with reduced risk of disease progression, as well as complications, including HCC development [286]. Moreover, several retrospective cohort studies and randomized controlled trials reported reduced HVPG and decreased risk of decompensation, HCC development and death in statin-treated patients with liver cirrhosis of different etiologies [287,288,289]. Finally, statins are described to exert beneficial effects on cardiovascular mortality and morbidity, that is especially of interest in patients with NASH [290].

Despite consistent data indicating potential anti-fibrotic effects, validity of these studies is limited due to retrospective design and lack of hard clinical endpoints, e.g., histological assessment of fibrosis. Moreover, considering drug-induced hepatotoxicity as a rare, though well-described side effect of statin as well as increased risk of rhabdomyolysis in patients with chronic liver disease due to impaired CYP3A4 metabolism in the liver, the safety profile of statins in patients with chronic liver disease and liver cirrhosis needs to be evaluated in detail. Thus, just recently Pose et al. reported rhabdomyolysis requiring treatment discontinuation in 19% (3/18) of patients with decompensated liver cirrhosis and treatment with 40mg simvastatin per day compared to 14% in 20mg simvastatin or placebo treated patients, respectively [291]. Another study reported severe rhabdomyolysis in 3% of patients with liver cirrhosis and statin use [289]. Taken together, growing experimental and clinical evidence suggest statins to exert beneficial pleiotropic effects on chronic liver disease progression and fibrosis. However, large prospective placebo-controlled trials with strong clinical endpoints as well as extended safety evaluation are awaited before recommendation of statins in patients with liver fibrosis (NCT03780673; NCT02968810; NCT04072601).

#### 4.2.5. Inhibition of HSC Activation

Numerous studies indicate Wnt/β-catenin signaling to be implicated in HSC activation and to contribute to liver fibrosis [169,292,293]. ICG-001 is a small molecule inhibitor that specifically disrupts the interaction between CBP and β-catenin. Initially developed for colon cancer therapy, ICG-001 [294] has been tested in several fibrosis studies and has been shown to inhibit TGF-β mediated upregulation of α-SMA and collagen 1 in mouse fibroblasts and human HSCs. Moreover, ICG-001 administration in a murine CCl_4_ induced mouse model of fibrosis attenuated HSC activation and ECM accumulation. Mechanistically, ICG-001 was found to affect macrophage infiltration and thereby reduce hepatic inflammation by affecting Wnt-dependent secretion of CCL12 by HSCs [295]. Apart from the liver, ICG-001 has also been reported to suppress pulmonary [296] and renal interstitial fibrosis [297].

As another member of CBP/β-catenin inhibitors, PRI-724 have been shown to inhibit HSC activation and collagen production in HCV transgenic mice [298]. Moreover, an independent study reported anti-fibrotic effects of PRI-724 in CCl_4_ induced murine liver fibrosis. In addition to confirmation of suppressed HSC activation, this study further indicated improved fibrosis resolution due to an increased F4/80+ CD11b+ and Ly6Clow CD11b+ macrophage population [11,299]. In a NASH mouse model, PRI-724 was shown to decrease hepatocyte apoptosis as well as fibrosis degree. Similar observations in CBP KO mice highlighted the CBP/β-catenin specific anti-fibrotic mode of action of PRI-724 [300]. A single-center, open label phase I clinical trial of PRI-724 in patients with HCV-associated liver cirrhosis showed dose dependent histological improvement (> 2 points decrease in histologic activity index score) in 3/12 patients, but deterioration by 2 points in 2/12 patients. A phase I/IIa clinical trial of PRI-724 in patients with hepatitis B or C related liver cirrhosis is expected to be completed in July 2020 and will further clarify the yet uncertain potential of PRI-724 in fibrosis treatment (NCT03620474).

FXR ligands have first been developed in the context of cholestatic liver diseases, as primary biliary cirrhosis. Thus, primary bile acids bind to FXR, that heterodimerizes with the retinoid X receptor, resulting in activation of its function as a transcription factor. FXR activation in hepatocytes and enterocytes hereby downregulates bile acid production, export as well as enteral and hepatic uptake. Moreover, it protects the intestinal mucosal barrier contributing to maintenance of the physiological gut microbiome and ultimately homeostasis of the liver-gut axis. FXR agonists such as obeticholic acid (OCA), may support reconstitution of gut microbiome composition, reduce bacterial translocation and inflammation [301,302]. Moreover, interfering the physiological feedback control system of bile acid production, synthetic FXR agonists as OCA have been developed and shown anti-cholestatic potency, leading to its approval for second-line treatment in PBC [303,304]. Recent clinical studies further indicate improvement of histological features, including fibrosis in patients with PBC after long-terms OCA treatment [305]. Moreover, FXR has been described to mediate inhibitory effects on HSCs activation [306]. Investigation of OCA in animal models of fibrosis further emphasized anti-fibrotic activity of FXR activation [306,307,308]. In 2015, the FLINT study, a phase 2b clinical trial reported histological improvement of fibrosis in NASH patients after short-term treatment with OCA for 72 weeks [309]. Just recently the first 18 month interim results of a multicenter, randomized placebo-controlled phase 3 clinical trial of long-term OCA treatment in NASH patients with fibrosis F1-F3 (NCT02548351) have been published and reports dose-dependent improvement of fibrosis in 23% of OCA 25 mg treated compared to 12% placebo treated participants. Moreover, OCA-treated patients showed less hepatocellular inflammation and ballooning. Reports regarding impact on non-invasive markers of fibrosis, long-term safety as well as clinical outcomes of this ongoing clinical trial (NCT02548351) are awaited in the future [310].

#### 4.2.6. Reduction of Fibrotic Scar evolution and Contractility

In liver cirrhosis up to 50% of the livers’ dry weight consists of collagens [311]. Collagen 1 (Col1) represents the most abundant collagen in fibrotic livers [312]. Jimenez et al. reported specific inhibition of Col1A1 siRNA containing lipoplexes in mouse models of liver fibrosis. Parenteral treatment hereby led to a 90% decrease in collagen production and 50% decrease of total collagen accumulation [313]. Another study on transgenic mice with inducible Col1 knockdown further reported additional anti-inflammatory effects [314]. Hsp47 is a Col1 chaperone and knockdown by siRNA can be used to block collagen synthesis. In order to target mainly fibrosis-effector cells, Sato et al. used Hsp47 siRNA containing vitamin A-coupled liposomes, which are predominantly uptaken by HSCs and achieved significant anti-fibrotic effects in 3 in-vivo models of liver fibrosis [315]. A clinical trial, investigating BMS 986263, an HSP47 siRNA delivering Lipid Nanoparticle, did not reveal any toxicity in healthy humans [316]. A phase 1b/2 open label dose escalation study of BMS 986,263 has recently been completed (NCT02227459). More studies on collagen inhibitors are expected to start in the next years.

Lysyl oxidases (LOXs), that are secreted by HSCs or MFs deamidate lysine or hydroxylysine residues in collagen or elastin and hereby crosslink collagen with each further [317,318]. These enzymes are therefore contributing to the stiffness of the ECM and impair degradation of deposited collagen fibrils by MMPs [319,320]. ECM stiffness in turn further promotes proliferation and activity of myofibroblasts via integrins [319,321]. LOX enzymes further exert functions on gene regulation [322], receptor function, and growth factor activity [323]. In fact, LOX enzymes impact on Collagen 3 expression [322]. Moreover, LOX members have been shown to oxidize PDGFRβ, thereby increasing the affinity to its ligand [324]. Development of liver fibrosis in a CCl_4_ based mouse model was shown to be accompanied by a 30-fold increase in LOX activity. Inhibition of all LOX members by β-aminopropionitrile decreased number and activity of MFs leading to a lower degree of liver fibrosis in this CCl_4_ induced liver fibrosis mouse model [325,326,327]. However, despite promising results in a mouse model of liver fibrosis [328], clinical trials investigating the LOXL2 blocking antibody Simtuzumab in patients with NASH, human immunodeficiency virus (HIV), or HCV-associated liver fibrosis as well as primary sclerosing cholangitis gave only disappointing results with no effect on liver fibrosis [329,330,331]. A later study showing rapid downregulation of LOXL2 after liver injury in contrast to stable upregulation of LOX and LOXL1, suggests a rather minor role of LOXL2 in liver fibrosis [317]. Future studies should address this observation by specific targeting of LOX o LOX1.

#### 4.2.7. Immune Modulation

Considering macrophages as the first pro-inflammatory response to liver injury [15,332,333], modulation of their first innate immune response represents a potential target for anti-fibrotic treatment approaches. Reduction of pro-inflammatory macrophage recruitment, using a dual CCR2/CCR5 inhibitor (Cenicriviroc) revealed anti-fibrotic effects in animal models of liver fibrosis [334,335,336]. Anti-fibrotic efficacy was also reported in a phase II clinical trial (CENTAUR; NCT02217475 [337]) of Cenicriviroc in NASH patients. In fact, especially patients with high disease activity and fibrosis stage benefit from oral Cenicriviroc treatment for 2 years. Surprisingly this was not accompanied by an anti-inflammatory activity [338]. Cenicriviroc was well tolerated, regardless of hepatic insufficiency. Headache and gastrointestinal disorders of mild severity were most frequent adverse events [338,339]. A phase 3 study on patients with advanced fibrosis and cirrhosis will further unravel the potency of CCR2/CCR5 inhibition for fibrosis therapy (AURORA; NCT 03028740).

Galectins are carbohydrate-binding proteins that get secreted by different cell types upon liver injury [340]. Extracellularly, these proteins bind to components of the ECM or to cell surface receptors [341,342]. Several studies indicate increased levels of galectin in inflammatory, fibrotic, or malignant liver tissue [343,344,345]. Due to its anti-apoptotic, cell differentiating and chemotactic properties, especially Gal-3, that is mainly secreted by activated macrophages, is involved in the pathophysiology of liver fibrosis [346,347,348]. Belapectin, an inhibitor or galectin-3 has shown potent anti-fibrotic efficacy in mouse and rat models of liver fibrosis [349,350] and was well tolerated in a phase 1 clinical trial [351]. However, just recently published results of a phase 2b placebo-controlled clinical study of belapectin in patients with NASH and liver fibrosis showed no effect on fibrosis following treatment for 52 weeks [352]. Still, considering significant protective effects on hepatocyte ballooning as well as significant lower HPVG and varices development in a subgroup of patients with NASH cirrhosis, a phase 3 clinical study in patients with NASH cirrhosis without varices at baseline timepoint is currently being initiated. The medication was well tolerated by NASH patients. Most frequently reported mild-moderate adverse events included infections, gastrointestinal, and musculoskeletal, as well as connective tissue disorders [352].

#### 4.2.8. Phytodrugs with Multi-Dimensional Effects on Liver Fibrosis

Several studies investigated herbal formulations and phytodrugs in treatment of liver fibrosis. Among many other phytochemicals, resveratrol, silymarin, and curcumin are the most extensively studied phytodrugs with potential anti-fibrotic activity [246,247].

Resveratrol is a natural antioxidant that can be found in a wide variety of plants. Frequently reported beneficial effects of resveratrol on health have been attributed to its mimicry of calorie restriction via activation of AMP-activated kinase (AMPK), nuclear factor (erythroid-derived)-like 2 (Nrf2), and nicotinamide adenine dinucleotide NAD+-dependent deacetylase (SIRT1) [353,354,355]. Treatment with resveratrol improves NASH and chronic liver disease in mouse models [264,353,356]. In NAFLD patients, a randomized, double-blinded clinical trial of oral resveratrol supplementation compared to placebo for 12 weeks revealed significant protective effects on markers of liver inflammation (serum level of alanine aminotransferase, NF-ĸB activity) and hepatic steatosis grade, but not on fibrosis [357].

Silymarin is an extract of the milk thistle (*Silybum marianum*), consisting of a mixture of different flavonoids and is applied as a supportive, hepatoprotective medication in patients with liver cirrhosis, chronic inflammatory, and toxic liver diseases since ages [358]. The consideration as a hepatoprotective agent is due to experimental data indicating potential prevention of hepatic injury by toxins and deceleration of fibrosis progression by the main ingredient, silibinin [359,360]. Moreover, long clinical experience exists for silymarin in prevention of alpha-amanitin-induced hepatotoxicity [361]. Thus, silibinin is regarded as a specific antidote of amanitin [362]. In terms of therapeutic application, small clinical studies reported anti-viral, anti-oxidative, anti-inflammatory, and insulin-sensitizing effects of silymarin in different etiologies of chronic liver disease, including ALD, NASH, and viral hepatitis [363,364,365,366].Thus, silymarin administration for four weeks reduced oxidative stress, fibrosis score, and activation of HSCs as well as Kupffer cells in a CCl_4_ based rat model of liver fibrosis [367,368]. However, in clinical practice low water solubility and limited oral bioavailability due to poor enteral absorption (23–47%) and high first-pass metabolism in the liver hamper use of silymarin [359,369]. Recently, new formulations of silymarin, including complexes with phosphatidylcholine and glyco-conjugates, have bypassed these limitations in oral application [370]. First studies using orally bioavailable silybin-vitamin E-phospholipids complexes for 12 months showed potential effects on hepatocyte ballooning, steatosis and liver fibrosis in 180 patients with NAFLD or NASH and 36 patients with HCV [371]. However, large double-blind, placebo-controlled studies of silymarin in treatment of chronic liver diseases are still missing, but needed to define its clinical value in not only supportive but also therapeutic applications [358].

Curcumin, the active compound of *Curcuma longa* have been investigated in several medical diseases and reported to exert tumor preventive, antiviral and anti-inflammatory effects in chronic liver disease [372,373]. Thus, curcumin administration inhibited hepatic inflammation, steatosis, fibrosis development, and progression in NASH in-vivo models [374,375]. Few clinical studies exist regarding the therapeutic potential of curcumin in chronic liver diseases. As observed for silymarin, curcumin is characterized by low oral bioavailability [376]. However, two independent randomized, placebo-controlled clinical trials reported decrease of biochemical and ultrasonographic markers of liver inflammation and steatosis by short-term curcumin administration (500-1000 mg/d) in 87 and 80 patients with NAFLD, respectively. Considering the low bioavailability of curcumin, these clinical effects are thought to be mediated by its metabolites [377,378]. Nevertheless, absent histological evaluation of changes following curcumin treatment strongly limits impact of the studies especially in terms of their anti-fibrotic capacity [379,380]. Moreover, a recent placebo-controlled clinical trial investigating lifestyle modification plus curcumin supplementation vs. placebo in 50 patients with NASH did not find significant advantages of curcumin in amelioration of biochemical and sonographic liver inflammation, steatosis, and fibrosis compared to lifestyle intervention alone [381]. Well-designed randomized placebo-controlled trials including histological examination are needed to define curcumin’s significance in clinical practice.

### 4.3. From Mouse to Men: Challenges in the Clinical Development of Anti-Fibrotic Compounds

The largely disappointing results of clinical phase 2 and 3 trials contrasts a long pipeline of promising anti-fibrotic candidate agents in preclinical models. This indicates the yet insufficient investigation or representation of disease biology by cell culture and animal models of fibrosis. Thus, conventional cell culture models of fibrosis do not recapitulate the multicellular and multidirectional evolution of fibrosis in humans. In fact, some agents have strong inhibitory effects on HSCs and myofibroblasts but mediate pro-fibrogenic mechanisms in other liver cells. Moreover, animal models of liver fibrosis have been shown to only partially reflect the human disease and reliable fibrotic readouts have long been undefined. In the past years, more and more guidelines for pre-clinical investigation and validation of potential anti-fibrotic agents have been proposed [24]. Thus, investigation of anti-fibrotic drugs in 2–3 validated and complementary fibrosis models are recommended. Widely accepted experimental approaches are CCl_4_ or Thioacetamide (TAA)-induced fibrosis, nutritional models mimicking NASH or biliary models [24,382,383]. Moreover, novel 3D in-vitro models that incorporate multiple parenchymal and non-parenchymal cell types as well as the fibrosis-driving fibrotic ECM itself are more and more established [20,384,385]. Consideration of the complex disease pathophysiology, implementation of complementary cell culture, and animal models of liver fibrosis as well as use of validated endpoints will hopefully revolutionize future anti-fibrotic opportunities.

## 5. Conclusions

Despite different mechanisms of primary liver injury, the progression of fibrotic liver disease follows shared patterns across the main liver disease etiologies. For all the etiologies, the development of hepatic fibrosis is initiated in response to hepatocytes or cholangiocytes damage, while progression of the fibrotic disease is mainly driven by dysregulated inflammatory processes. Thus, chronic viral infection triggers robust immune responses leading to chronic inflammation and hepatocyte death. The progression of ALD and NASH is marked by the accumulation of fat in the liver leading to hepatocyte apoptosis and oxidative stress. Repetitive peaks of inflammation, followed by anti-inflammatory, reparative immune responses activate collagen-producing myofibroblasts that account for excessive accumulation of ECM, the cellular correlate of tissue fibrosis. Removal or elimination of the initial trigger such as viral cure may slow down or reverse liver fibrosis, but mostly occurs often too slowly or too infrequent to avoid life-threatening complications in particular in late-stage disease. While many anti-fibrotic candidate agents have shown robust effects in experimental animal models, their anti-fibrotic effects in clinical trials are less clear. The fact that selected anti-fibrotic agents have shown evidence for potential effect on fibrosis progression in clinical trials, suggests that it is possible to target liver fibrosis by pharmacological intervention. However, additional clinical studies are needed to confirm the long-term impact and robustness of these findings. Given the still limited clinical efficacy and adverse effects of the current compounds in clinical development, there is a high unmet medical need for more efficient and safe anti-fibrotic drugs to significantly improve the patients’ outcome. The recent development of innovative patient-derived models for liver fibrosis may advance the development of compounds with anti-fibrotic properties in the future.

## Figures and Tables

**Table 1:** Examples for compounds in clinical development aiming to reduce fibrosis by inhibition of hepatocyte apoptosis and reduction of oxidative stress.

Anti-fibrotic Mechanism	Agent	Rationale	Molecular Mode of Action in Preclinical Studies	Key Findings in Clinical Trials
Inhibition of hepatocyte apoptosis	Pan-caspase inhibitor Emricasan	Hepatocyte apoptosis is a major trigger of inflammation and HSC activation [249,250].	Decreased HSCs activation and improvement of liver function in rat CCl_4_ model [253].	Phase 2: Improvement of liver inflammation or fibrosis and tendency towards worsening of hepatocyte ballooning in NASH patients with F1-F3 fibrosis [254]. Small reductive effect on HVPG in cirrhotic NASH patients [255]. No effects in patients with acutely decompensated cirrhosis [256]. NCT02138253: clinical trial of Emricasan in the setting of post-transplant HCV-induced fibrosis after SVR: awaited 2020.
	ASK1 inhibitor, selonsertib	Mediation of hepatocyte apoptosis via activation of JNK and p38 MAP kinases [257].	Improvement of steatosis and fibrosis in NASH mouse model [257].	Phase 2: Improvement of histological degree of fibrosis in patients with NASH F2-3 [260] Decrease of liver stiffness by MRE and improvements of non-invasive markers of fibrosis and inflammation [386]. Phase 3: STELLAR-3 and 4: Selonsertib in NASH patients and bridging fibrosis or cirrhosis: ongoing (NCT03053050; NCT03053063)
Reduction of oxidative stress	Natural antioxidant with several targets, Resveratrol	Anti-inflammatory and antioxidant activity	Resveratrol reduces inflammation, fibrosis [264] as well as steatosis [387] in a mice models of NASH.	Phase 2: significant protective effects of resveratrol on markers of liver inflammation and hepatic steatosis grade within 12 weeks of treatment, no effect on fibrosis [357].
	Dual NOX1/4 inhibitor, GKT137831	Activation of HSCs (NOX1) and induction of apoptosis in hepatocytes (NOX4) by production of superoxide radicals [155,266].	Anti-fibrotic effect in CCl_4_ and bile duct ligation based mouse models of liver fibrosis via suppression of ROS production in HSCs in-vitro and in-vivo [267].	Phase 2: significant effects on serological cholestasis parameters after 6 weeks of treatment in PBC. Ongoing study (NCT03226067).

**Table 2:** Examples for compounds in clinical development aiming to reduce fibrosis by inhibition of HSC activation and reduction of fibrotic scar evolution.

Anti-fibrotic Mechanism	Agent	Rationale	Molecular Mode of Action in Preclinical Studies	Key Findings in Clinical Trials
Inhibition of HSC activation	FXR agonist, Obeticholic acid	Transcriptional regulation of fibrogenic genes in HSCs [306]. Improvement of intestinal mucosal barrier and homeostasis of gut-liver axis [301,302].	Downregulation of collagen 1 synthesis in HSCs, potent anti-fibrotic effect in animal models of liver fibrosis [306].	Phase 2: Improvement of fibrosis after 72 weeks treatment with OCA [309] Phase 3 (CENTAUR): dose-dependent improvement of fibrosis in 23% of OCA 25 mg treated compared to 12% placebo treated participants. Reduction of hepatocellular inflammation and ballooning [310].
	CBP/β-catenin small molecule inhibitor PRI-724	Implication of Wnt/β-catenin signaling in HSC activation and liver fibrosis [169,292,293].	Inhibition of HSC activation in HCV transgenic mice as well as CCl_4_ based murine liver fibrosis [298]. Beneficial effects on fibrosis resolution by activating anti-fibrotic macrophage subpopulations [299]. Decrease of hepatocyte apoptosis as well as fibrosis degree in NASH mouse model [300].	Phase 1: dose dependent histological improvement (>2 point decrease in histologic activity index score) in 3/12 patients, but deterioration by 2 points in 2/12 patients with HCV associated cirrhosis [388]. Phase 2: PRI-724 in patients with hepatitis B or C related liver cirrhosis: expected to be completed in July 2020 (NCT03620474).
Reduction of fibrotic scar evolution and contractility	Hsp47 siRNA delivering lipid nanoparticle, BMS 986263	Function of Hsp47 as a collagen 1 chaperone [315].	Significant anti-fibrotic effects in 3 and in-vivo models of liver fibrosis [315].	Phase 1b/2: open label dose escalation study of BMS 986,263 in patients with moderate to severe fibrosis: completed, not yet published (NCT02227459).
	LOXL2 specific monoclonal antibody, AB0023 (Simtuzumab)	Contributing of LOXL2 to ECM stiffness and hampered degradation of deposited collagen fibrils [317,318,319,320] Implication in Collagen 3 expression [322] and PDGFR sensitivity [324].	Potent anti-fibrotic activity in bleomycin based mouse model of liver fibrosis via inhibition of collagen-crosslinking and its downstream activating effect on TGF-β1 signaling that contributes to myofibroblast simulation [328].	Phase 2: No effect on fibrosis in NASH, PSC, or patients with HIV and/or HCV-infected patients with liver fibrosis [329,330,331].

**Table 3:** Examples for compounds in clinical development aiming to reduce fibrosis by immune modulation.

Anti-fibrotic Mechanism	Agent	Rationale	Molecular Mechanism of Action in Preclinical Studies	Key Findings in Clinical Trials
Immune modulation	CCR2/CCR5 inhibitor, Cenicriviroc	Involvement of CCR2/CCR5 mediated monocyte and macrophage recruitment during early pro-fibrogenic response [15,332,333].	Dose-dependent decrease in monocyte/macrophage recruitment [334,335,336].Significant decrease in lobular inflammation, hepatocellular ballooning as well as collagen 1 and α-SMA protein expression in NASH mouse model [334].	Phase 2: Improvement of fibrosis stage (> 1 stage) without worsening of steatohepatitis especially in patients with high disease activity (NAS > 5, prominent hepatocyte ballooning, F2-F3 fibrosis) [338]. No effect on lobular inflammation, but decrease in serological markers of systemic inflammation (hsCRP, IL6, fibrinogen) [338]. Phase 3: AURORA, NASH patients with advanced fibrosis and cirrhosis (NCT03028740): ongoing
	Inhibitor of galectin-3, Belapectin	Function of galectin-3 as a chemoattractant for macrophages and monocytes, hereby accelerating further pro-inflammatory and pro-fibrogenic immune responses [347,348]. Activator of MMP2 and MMP9 [342].	Dose-dependent reduction of NAS, fibrosis and portal pressure in rat and murine models of NASH potentially due to an impact on macrophage polarization and reduced activation of HSCs [349,350].	Phase 2: No effect on fibrosis following within 52 weeks of treatment in NASH patients. Significant protective effects on hepatocyte ballooning as well as significant lower HPVG and varices development in a subgroup of patients with NASH cirrhosis [352].
